# Incidence and Risk Factors of Surgical Site Infection in 376 Mastectomy Procedures in Female Dogs: A Retrospective Cohort Study

**DOI:** 10.3390/vetsci12060553

**Published:** 2025-06-05

**Authors:** Manuel Fuertes-Recuero, Silvia Penelo, María Suarez-Redondo, Alba Eceiza-Zubicaray, Mario Arenillas, Guillermo Valdivia, Paula García San José, Laura Peña, Dolores Pérez-Alenza, Gustavo Ortiz-Díez

**Affiliations:** 1 Complutense Veterinary Teaching Hospital, Complutense University of Madrid, Avda. Puerta de Hierro s/n, 28040 Madrid, Spain; manufuer@ucm.es (M.F.-R.); spenelo@ucm.es (S.P.); marsuare@ucm.es (M.S.-R.); albaecei@ucm.es (A.E.-Z.); marioare@ucm.es (M.A.); edgargva@ucm.es (G.V.); pgsanjose@ucm.es (P.G.S.J.); mdpa@vet.ucm.es (D.P.-A.); 2 Department of Physiology, Veterinary Medicine School, Complutense University of Madrid, Avda. Puerta de Hierro s/n, 28040 Madrid, Spain; 3 Department of Animal Medicine and Surgery, Veterinary Medicine School, Complutense University of Madrid, Avda. Puerta de Hierro s/n, 28040 Madrid, Spain

**Keywords:** mammary tumours, postoperative complications, multimodal analgesia, canine surgery

## Abstract

The incidence of surgical site infection (SSI) following mastectomy in female dogs remains poorly characterized, despite its relevance to postoperative outcomes and patient welfare. This retrospective cohort study aimed to determine the incidence of SSI and identify associated clinical and surgical risk factors in dogs undergoing mastectomy procedures at a veterinary teaching hospital between 2013 and 2022. SSI diagnosis was based on the Centers for Disease Control (CDC) criteria and identified structured passive surveillance of medical records. Univariate logistic regression was carried out to screen for potential risk factors, followed by multivariate analysis of selected variables. Among 376 mastectomy procedures performed in 306 dogs, 33 SSIs were detected, resulting in an overall incidence of 8.8% (95% CI: 6.1–12.1). The majority were superficial infections (93.9%), with only two cases classified as deep SSI; no organ/space infections were recorded. Prolonged anaesthesia duration (*p* = 0.041) and intraoperative hypothermia (*p* = 0.026) were independently associated with increased SSI risk. These findings indicate that the incidence of SSI after canine mastectomy is comparable to that reported for other clean surgeries. Prolonged anaesthesia and hypothermia represent modifiable risk factors, highlighting the importance of perioperative temperature control and optimized anaesthetic management. No significant associations were observed with patient-related variables, mastectomy type, or wound soaker catheter use, supporting its safe inclusion in multimodal analgesia.

## Simple Summary

Surgical site infections are complications that may occur after surgery and can impact the recovery and welfare of animals. This study is focused on female dogs undergoing mastectomy, a surgical procedure to remove the tumours of the mammary glands. We reviewed the medical records of 306 dogs that underwent 376 mastectomies at a veterinary teaching hospital over a nine-year period, looking for cases of postoperative infection. Infections were found in 33 of these procedures (8.8% of the total). Most of the infections were superficial, while only two cases affected the muscular fascia. Prolonged anaesthetic duration and intraoperative hypothermia were also found to be risk factors. However, other factors, such as the type of mastectomy or the use of special catheters for pain relief, did not increase the risk. These results suggest that proper management of body temperature and efficient use of anaesthesia can reduce complications and aid in better recovery for dogs. This information can potentially help veterinary professionals improve care during surgery and promote a faster, safer recovery for dogs undergoing mastectomies.

## 1. Introduction

Mammary tumours account for 50% to 70% of all tumours in intact female dogs [1,2,3,4,5]. The prevalence is variable among countries, being greater in countries where ovariectomy is not routinely performed [6]. Mammary tumours in dogs represent a wide spectrum of histological subtypes with diverse biological behaviour [7,8,9]. Surgical excision is the treatment of choice for the majority of the cases, including simple, regional, or radical mastectomy [10], with the decision influenced by factors such as metastatic status, and the number and size of nodules [7,8].

Surgical site infection (SSI) is a common complication following any surgical procedure, including mastectomies [11], that can increase morbidity, mortality, and overall treatment cost [12,13,14,15,16,17,18,19,20]. Several studies conducted in dogs and cats undergoing surgical procedures, specifically evaluating SSIs, have described an overall incidence of 3.0–8.7% [13,14,15,17,18,19,20]. These studies were either prospective or retrospective and included different types of surgery (soft tissue procedures, orthopaedic surgery, etc.) making it difficult to standardize and compare them [21,22]. To address this gap, studies are increasingly focusing on the incidence of SSI and the risk factors associated with a specific type of surgery [23,24,25]. Specially, the incidence of SSIs in mastectomy procedures has been reported as 8.9% [11], while other studies have noted rates as high as 11.0% and 66.7% without applying the Center for Disease Control (CDC) criteria [11,26,27].

Risk factors previously associated with different clean surgical procedures include the duration of anaesthesia, surgical time, and the presence of a concomitant endocrine disease [12,17,18]. The only risk factor described in dogs for developing SSI in mastectomy procedures is the number of mammary glands removed, with dogs undergoing excision of two or more glands at a higher risk [11].

Subcutaneous infiltration with continuous local anaesthetic delivered through analgesic diffusion catheters, also called wound soaker catheters (WSC), as part of the postoperative multimodal analgesic plan has been described in both human and veterinary medicine. In dogs, WSCs have been used to control postoperative pain following limb amputation, muscle dissection, and total ear canal ablation [28,29,30,31,32]. Despite their potential benefits, the use of WSCs has only been described in 14 dogs following mastectomy [26,33,34]. Although it has been postulated that complications associated with WSCs may include premature catheter removal by the patient, catheter occlusion, seroma, and SSI [28,32], no association between WSCs and increased SSI risk has been investigated in veterinary medicine [28]. A recent case series did not find bacterial growth when WSCs were analysed after use during mastectomies of 11 female dogs [34].

The incidence of mammary tumours in dogs is high in southern Europe, in countries such as Spain, where spaying young female dogs is not a widespread practice [3,35,36,37]. Therefore, due to the high case load of mastectomies performed in Spain, the purposes of the study were (1) to describe the incidence of SSI in patients undergoing mastectomy and (2) to identify potential risk factors associated with SSI in mastectomies carried out at the Veterinary Teaching Hospital (VTH). This study is the first to evaluate the potential role of wound soaker catheters (WSCs) in the development of SSI during mastectomy, an issue that has not been previously explored in the veterinary surgical literature.

## 2. Materials and Methods

A retrospective cohort study was performed using the records of female dogs that underwent surgical removal of mammary tumours, ranging from a single gland mastectomy to unilateral total mastectomy, between 1 September 2013 and 31 December 2022 at the VTH. Dogs of all breeds, ages, weights, and reproductive statuses were included in the study. Patients who had undergone previous mastectomies were included. Patients receiving concurrent therapy for another systemic disease were included. Patients with missing information about anaesthesia, surgery, and wound healing in their medical records for at least the first month postoperatively were excluded from the study. Dogs undergoing surgeries other than mammary tumour resection during the same anaesthetic procedure, except for ovariohysterectomy, were also excluded from the study. Ethical review and approval were not required for this retrospective study, as it involved only analysis of anonymized clinical data in accordance with local legislation. Written informed consent for the use of clinical data for research purposes was obtained from the owners upon admission. All data were handled confidentially to ensure patient and owner privacy.

All dogs were managed according to a rigorous, standardized multidisciplinary protocol established by the Mammary Oncology Unit (MOU) of the VTH (Appendix A.1), which encompassed clinical diagnosis, tumour staging, surgical management, histopathological evaluation, and structured postoperative follow-up [38,39,40].

Preoperative clinical diagnosis and staging included haematology, serum biochemistry, thoracic radiography, and tumour staging following previously established criteria [41]. All mammary glands were meticulously palpated by at least one nationally accredited specialist in veterinary oncology of MOU, and the dimensions and anatomical locations of detected masses were systematically measured using callipers and documented. Preoperative skin antisepsis was performed following a standardized protocol, combining the immediate bactericidal activity of alcohol with the residual effect of chlorhexidine. Initially, the surgical site was cleansed with a single application of 70% isopropyl alcohol. Then, three sequential applications of 2% chlorhexidine gluconate were applied, each left in contact with the skin for approximately 30 s and removed with clean gauzes. After the final application, the area was left to dry, and the solution remained in contact for at least 1 min prior to draping. Immediately before surgical incision, any residual solution was gently removed using sterile gauzes. This protocol follows validated veterinary asepsis practices [42].

Surgery was performed by personnel nationally accredited in veterinary surgery or by trainees under the direct supervision of senior surgical staff. In intact female dogs, ovariohysterectomy was routinely performed immediately prior to mastectomy. Surgical resection of tumours was conducted following standard oncological guidelines, achieving surgical margins typically ranging from 1 to 3 cm depending on tumour size. The type of mastectomy performed was categorized according to the criteria defined by the MOU as simple mastectomy (removal of one gland), regional mastectomy (removal of ≥2 adjacent glands), or radical mastectomy (removal of an entire mammary chain) [38]. Inguinal lymph nodes were routinely excised during removal of inguinal mammary glands, while axillary lymph nodes were resected only if enlarged or if cytological results were suggestive of metastatic involvement. In non-spayed females undergoing ovariohysterectomy, the abdominal wall was closed using a continuous pattern with absorbable monofilament polydioxanone sutures (sizes 2/0 to 1, depending on body size). The subcutaneous tissue at the mastectomy site was closed in two layers: first, interrupted approximating sutures, followed by a continuous subcutaneous pattern, both with absorbable monofilament glyconate sutures (sizes 2/0 and 3/0, respectively). The skin was closed using cruciate interrupted sutures with 3/0 nylon or surgical staples, at the surgeon’s discretion. When wound soaker catheters were placed, they were positioned in the subcutaneous space over the abdominal fascia prior to closure. Postoperatively, sterile gauze pads and tubular mesh bandages were routinely applied to protect the surgical site and secure the catheter in place. The dressing was removed upon catheter withdrawal, typically 48 to 72 h after surgery. In selected cases, such as when wound complications were suspected or when patient behaviour posed a risk to wound integrity, dressings were maintained until suture or staple removal.

Dogs with extensive bilateral mammary disease underwent staged procedures with an interval of 1 to 2 months between surgeries, depending on postoperative complications, owner availability, and patient recovery following the first mastectomy. Each surgery was recorded and analysed independently.

During anaesthetic induction, intravenous cefazolin (Cefazolina Normon^®^ 22 mg/kg) was administered and repeated every 120 min until completion of the surgical procedure. Postoperative antibiotic use was determined at the discretion of the attending surgeon. Excised tissues were routinely submitted for histopathological analysis performed by board-certified veterinary pathologists.

A structured postoperative follow-up protocol was consistently implemented. Dogs were monitored by personnel nationally accredited in veterinary surgery at least twice after surgery (between postoperative days 3–5 and 12–15). Additional evaluations were scheduled as necessary if complications arose. Sutures were removed at the final follow-up appointment (12–21 days postoperatively). Long-term follow-up intervals were individualized based on histopathological findings and the potential need for further treatments, in accordance with the established guidelines of the MOU.

Both general and postoperative data were collected from the medical records of the hospital. The independent variables were classified as patient-related, surgical, or postoperative variables.

Patient-related variables included age, breed, reproductive status, weight, body condition score (from one to nine according to the criteria of the World Small Animal Veterinary Association, Global Nutrition Committee), haematology and/or biochemistry abnormalities (defined based on the reference intervals routinely used in our institution, as established for the analytical systems employed in our clinical pathology laboratory), concurrent systemic or organ diseases, American Society of Anesthesiologists (ASA) status classification, type of medical treatment at the time of surgery, and the performance of anaesthetic locoregional blocks.

Surgical variables included surgeon’s experience, type of mastectomy (simple, regional, or radical), anatomical localization of excised glands, number of mammary tumours, location and maximum diameter of the tumours, length of anaesthesia (defined as the time from anaesthetic induction to extubation), length of surgery (defined from the first surgical incision to placement of the last suture), placement of WSC into the subcutaneous space, minimum temperature during surgery, and episodes of intraoperative surgical complications. Anaesthetic complications were also recorded and included intraoperative hypothermia (defined as any recorded instance of body temperature falling below 36.5 °C during surgery, as measured via continuous temperature monitoring throughout the anaesthetic procedure) [43], hypotension (mean arterial pressure < 60 mmHg) [19], nociception (requiring intraoperative administration of fentanyl), and bradycardia (heart rate < 40 bpm).

Postoperative variables included the administration of antibiotics, anti-inflammatory drugs, length of hospital stay (days), and complications other than SSI (reintervention, hernia, seroma, hematoma, pain, swelling, redness, and subcutaneous emphysema). Pain was assessed postoperatively by trained veterinary surgeons using a composite pain scale adapted from the Glasgow Composite Measure Pain Scale-Short Form (CMPS-SF), which includes evaluation of posture, activity, response to palpation, and vocalization. Presence of pain was considered when the score was 6/24 or higher and recorded as ‘Yes’ or ‘No’ [44].

For each surgical procedure, in cases with multiple tumours present in the same mammary chain, the tumour with the highest clinical stage (tumour size) was selected for statistical analysis. Excised tumours were classified as benign or malignant, and malignant tumours were further categorized as carcinomas or other malignant tumour types.

SSI was defined according to CDC criteria, which include superficial, deep, or organ/space infection, occurring within 30 days of the surgical procedure, characterized by local signs such as erythema, heat, swelling, or purulent discharge, or confirmed by microbial culture [45,46]. Surgical site infections (SSI) were defined according to CDC criteria and classified as follows: (1) superficial incisional, involving only the skin or subcutaneous tissue of the incision; (2) deep incisional, involving the deeper soft tissues such as fascia and muscle; or (3) organ/space, involving any part of the body opened or manipulated during surgery that lies beneath the fascial plane [45,46]. A passive surveillance system was used. Postoperative evaluations were performed by trained surgeons on days 3–5 and 12–15, and additionally when complications were suspected. Treatment of the SSI with the administration of postoperative antibiotics was based on the surgeon’s preference.

The incidence of SSI was calculated per 100 surgical procedures using the following formula: (number of SSI cases/total number of surgical procedures) × 100. Results are expressed as percentages with their corresponding 95% confidence intervals (CI), calculated by the exact binomial method.

Data distribution was assessed using the Kolmogorov–Smirnov test. Categorical variables were summarized as frequencies. Continuous variables were presented as mean and standard deviation (SD) if normally distributed, or as median and interquartile range (IQR; Q1–Q3) if normality assumptions were not met.

Associations between independent variables and SSI (dependent variable) were initially evaluated by univariate logistic regression analysis. To simplify interpretation, quantitative variables (weight, body condition score, age, number of mammary tumours, maximum tumour diameter, duration of surgery and anaesthesia) were categorized into two groups based on their median values. For intraoperative temperature, categorization was based on a clinically relevant cut-off (<36.5 °C), in line with established definitions of mild hypothermia in veterinary anaesthesia [43]. Variables showing a *p*-value < 0.100 in the univariate analysis were subsequently included in a multivariate logistic regression model, using a forward stepwise selection method. Effects of the variables included in the final model are presented as odds ratios (OR) with their corresponding 95% CI. The goodness-of-fit of the final model was assessed using the Hosmer–Lemeshow test, and its discriminative ability was evaluated by receiver operating characteristic (ROC) curve analysis, reporting the area under the curve (AUC), with corresponding 95% CI and *p*-value. Statistical significance was set at *p* < 0.050. Statistical analyses were performed using IBM SPSS software for Windows (version 28.0).

## 3. Results

### 3.1. Epidemiological and Clinical Preoperative Findings

A total of 306 female dogs underwent mastectomy. Seventy (22.9%) dogs required two separate procedures performed 1 to 2 months apart, depending on postoperative recovery, resulting in a total of 376 mastectomies analysed independently. The most common breed was mixed breed (98; 32.0%), followed by Yorkshire Terrier (37; 12.1%) and German Shepherd (17; 5.6%) (Table A1).

For the 376 mastectomy procedures, median weight at surgery was 10 kg (IQR: 5.7–22.7 kg; range: 1.6–50 kg). Median body condition score (BCS) was 5 (IQR: 4–6). Median age at surgery was 10 years (IQR: 8.2–11.6; range: 2.6–16.6 years). Concurrent diseases were identified in 25 (6.6%) procedures, including skin diseases (11; 2.9%), leishmaniosis (9; 2.3%), and endocrine disorders (5; 1.3%). Based on ASA status, 357 (94.9%) procedures were classified as ASA 2, and 19 (5.1%) as ASA 3.

Haematological abnormalities were identified in some procedures, including thrombocytosis (11; 2.9%), anaemia (8; 2.2%), leukopenia (4; 1.0%), leucocytosis (4; 1.0%), and thrombocytopenia (4; 1.0%). Biochemical abnormalities included hyperproteinaemia (18; 4.8%), hypoproteinaemia (4; 1.0%), azotaemia (3; 0.8%), hyperglycaemia (2; 0.5%), and hypalbuminaemia (2; 0.5%). Locoregional anaesthetic blocks were administered in 106 (29.3%) procedures, including transversus abdominis plane (TAP) blocks (58; 54.7%) and epidural blocks (48; 45.3%).

### 3.2. Surgical Findings

Senior surgeons performed 331 (88.0%) procedures, while surgeons-in-training performed 45 (12.0%) under direct supervision. Radical mastectomy was the most frequent surgical approach (192; 51.4%), followed by regional mastectomy (155; 41.5%) and simple mastectomy (26; 6.9%). Concurrent ovariohysterectomy was performed in 218 (58.4%) procedures.

The median number of mammary tumours per procedure was 2 (IQR: 1–4; range: 1–12). The median maximum tumour diameter was 1.5 cm (IQR: 0.96–3.0 cm; range: 0.1–18 cm). The mean duration of surgery was 106 min (SD: 45.8; range: 15–267 min). The mean duration of anaesthesia was 173.5 min (SD: 54.6; range: 28–396 min).

Intraoperative complications occurred in 178 (47.2%) procedures, including nociception (123; 33.7%), hypotension (119; 32.5%), and bradycardia (54; 14.8%). Hypothermia (temperature < 36.5 °C) occurred in 46 (12.5%) procedures, with a mean minimum temperature of 36.1 °C (SD: 1.0 °C). Moderate intraoperative bleeding beyond expected occurred in 4 (1.1%) procedures; all were resolved without further complications. Wound soaker catheters (WSCs) were placed intraoperatively in 210 (55.9%) procedures.

### 3.3. Postoperative Findings

Surgical site infection (SSI) was identified in 33 (8.8%) procedures. Other postoperative complications (excluding SSI) occurred in 113 (31.0%) procedures, including haematoma (50; 13.4%), seroma (40; 12.0%), vomiting (12; 3.2%), diarrhoea (6; 1.6%), wound necrosis (4; 1.0%), and subcutaneous emphysema (1; 0.3%).

Antibiotics were administered postoperatively in 327 (93.6%) procedures, primarily amoxicillin-clavulanic acid (210; 60.5%) or cefazolin (109; 31.4%). The median length of hospital stay was 0 days (IQR: 0–1 day; range: 0–13 days). The median time until surgical discharge (defined as complete wound healing and no further need for surgical follow-up) was 13 days (IQR: 11–15 days; range: 7–57 days).

In 184 out of the 376 mastectomies (49.3%), at least one malignant tumour was diagnosed. Mammary carcinomas accounted for 181 of the malignant tumours (98.4%), while single cases of osteosarcoma, malignant myoepithelioma, and undifferentiated sarcoma were also diagnosed. The histological grades of carcinomas were grade I (117; 31.1%), grade II (39; 10.4%), and grade III (25; 6.6%). The descriptive results are presented in Table 1.

### 3.4. Incidence of Surgical Site Infection

Of the 376 procedures analysed, SSI was diagnosed in 33 (8.8%; 95% CI: 6.1–12.1%). The vast majority were classified as superficial (31/33; 93.9%), with only two cases (6.1%) meeting the criteria for deep infection; no organ/space SSIs were identified. Notably, none of the dogs that underwent two separate mastectomy procedures developed SSI after either surgery.

### 3.5. Risk Factors for Surgical Site Infection: Univariate and Multivariate Analysis

Univariable analysis identified two surgical variables significantly associated with surgical site infection (SSI): anaesthesia duration > 173.5 min (OR 2.4, 95% CI: 1.1–5.2; *p* = 0.024) and intraoperative hypothermia (OR 2.9, 95% CI: 1.1–7.8; *p* = 0.032) (Table A2).

In multivariable analysis, both variables remained independently associated with increased SSI risk: anaesthesia duration > 173.5 min (adjusted OR 2.2, 95% CI: 1.0–5.2; *p* = 0.041) and hypothermia (adjusted OR 3.1, 95% CI: 1.1–8.3; *p* = 0.026). The model showed good calibration (Hosmer–Lemeshow test, *p* = 0.888) and moderate discriminatory ability (AUC 0.657, 95% CI: 0.564–0.750) (Table 2).

## 4. Discussion

This study reports an overall incidence of surgical site infection (SSI) of 8.8% in 376 mastectomy procedures in dogs, a rate comparable to previous studies evaluating clean surgical interventions in veterinary medicine [12,18]. Notably, our findings identify prolonged anaesthetic duration and intraoperative hypothermia as independent, modifiable risk factors significantly associated with increased SSI risk. These results underline the importance of optimizing perioperative management, specifically through improved thermal regulation and efficient anaesthetic protocols, to minimize postoperative complications. Additionally, neither the extent of mastectomy nor the use of wound soaker catheters (WSCs) significantly influenced SSI incidence, providing valuable clinical information to support their safe implementation in multimodal analgesic strategies. The robustness of these conclusions is supported by the large sample size and the consistent application of standardized clinical, surgical, histopathological, and follow-up protocols.

The SSI incidence identified in this study (8.8%) is consistent with previous veterinary reports evaluating canine mastectomies using standardized CDC criteria [11,26]. For instance, a retrospective study analysing 135 canine mastectomies reported an almost identical SSI rate (8.9%) in a smaller cohort (*n* = 135) predominantly undergoing simple or regional mastectomies [11]. Conversely, Evans et al. (2021) found a slightly higher incidence (11%), although this figure was derived from diagnostic criteria based solely on clinical signs, bacteriology, and cytology, rather than standardized CDC definitions, which might have resulted in variability in infection detection [26].

Other veterinary studies applying CDC criteria have reported lower SSI incidences, ranging between 4.8% and 5.9%, but these involved diverse soft tissue procedures [12,17,18]. Differences in surgical complexity, patient characteristics, and postoperative monitoring methods inherently limit comparability between these studies and ours. For example, previous research highlights that structured active surveillance post-discharge increases the reported SSI rates, detecting up to 27.8% more infections compared to passive surveillance [22]. Thus, recent veterinary and human medical literature increasingly emphasizes assessing SSI risk within homogeneous surgical populations to minimize methodological bias [17,25]. The use of standardized CDC criteria, combined with a structured follow-up and a larger homogeneous sample, facilitates a more reliable interpretation of SSI incidence specifically within canine mastectomy populations.

### Risk Factors

Our results identify intraoperative hypothermia as a significant risk factor for SSI in dogs undergoing mastectomy. Previous veterinary studies investigating intraoperative hypothermia as a risk factor for surgical site infection (SSI) have produced inconclusive results, often due to methodological limitations. For instance, two studies that specifically evaluated this association [12,14] did not find significant relationships, likely influenced by the routine use of active warming strategies such as warm-water blankets. Additionally, broader studies on SSI have seldom recorded intraoperative temperature consistently [17,18], which limits comparability. In contrast, our study systematically monitored temperature and identified intraoperative hypothermia (defined as <36.5 °C) in 12.5% of procedures, despite the use of forced-air warming systems. Hypothermia was independently associated with a significantly increased risk of SSI, with an adjusted odds ratio of 3.1 (95% CI: 1.1–8.3), indicating that affected dogs were approximately three times more likely to develop postoperative infection. These findings are consistent with the human medical literature linking even mild perioperative hypothermia to increased SSI risk [47,48,49,50] and suggest that additional strategies beyond forced-air warming may be required to ensure adequate thermal control during surgery.

Our findings indicate that prolonged anaesthetic duration is associated with an increased risk of SSI, a result consistent with previous veterinary and human studies reporting similar associations across various surgical procedures beyond mastectomy [12,15,17,25,51]. This relationship could be explained by several mechanisms, including extended exposure of surgical incisions to environmental contaminants in the operating theatre, which might increase bacterial colonization [52] or immunosuppressive effects associated with prolonged anaesthetic administration [53]. Therefore, based on these results, minimizing anaesthetic duration should be strongly encouraged. Effective coordination between surgical and anaesthetic teams is essential to achieving this goal. Additionally, structured surgical and anaesthetic checklists might further optimize procedural efficiency, potentially reducing operative time and postoperative complications [54].

This is the first veterinary study to specifically evaluate wound soaker catheter (WSC) placement as a potential risk factor for surgical site infection (SSI) in dogs undergoing mastectomy. No association was observed. Previous reports describing WSC use in mastectomies [33,34] and other clean surgeries such as amputations and ear canal ablation [28,32] have similarly reported low complication rates and no consistent evidence of increased SSI risk. In our study, WSCs were placed subcutaneously and used to administer bupivacaine 0.25% (1–2 mL every 6–8 h for up to 72 h), following protocols comparable in dose, frequency, and catheter dwell time to those reported in the veterinary literature. Notably, a recent case series involving 11 dogs undergoing mastectomy with WSC placement found no evidence of bacterial contamination associated with the catheters [34]. Bupivacaine has shown antimicrobial activity in vitro against *Staphylococcus aureus*, *E. coli*, and other pathogens relevant to postoperative infection [55], although its clinical relevance in vivo remains uncertain and likely depends on factors such as tissue penetration and local drug concentration. Therefore, while a contribution from bupivacaine cannot be excluded, the absence of increased SSI risk in our cohort is more likely the result of multiple factors, including limited catheter duration, consistent surgical technique, and adherence to aseptic protocols. These observations align with other veterinary case series in which WSCs were well tolerated and not associated with increased postoperative complications [28,32,33,56,57,58]. Regarding the extent of surgery, our study found no difference in SSI risk between regional mastectomy (involving smaller incisions) and radical mastectomy (involving larger incisions). This contrasts with previous research, where radical mastectomies have been associated with a higher risk of postoperative complications, including SSI [27]. Additionally, earlier studies had indicated that dogs undergoing removal of two or more mammary glands had an increased risk of SSI and other postoperative complications compared to those undergoing removal of a single gland [11]. This could be explained by the greater surgical trauma associated with larger wounds, increased suture tension, and deeper dead space, factors known to impair healing and predispose patients to SSI [59,60]. However, in our study, the extent of surgery was not identified as a risk factor for SSI. This discrepancy with earlier findings might be related to our routine use of bupivacaine administered through wound soaker catheters (WSCs), given its known antimicrobial effects [55]. Although tumour malignancy has previously been associated with increased postoperative complications in various types of canine neoplasia [61,62], our results did not identify tumour malignancy as a significant risk factor for SSI, in line with another recent study [25]. This lack of association may reflect the greater influence of surgical and perioperative management factors compared to tumour-specific characteristics in the development of SSI.

The use of perioperative antimicrobial prophylaxis in clean surgical procedures, such as mastectomies, remains controversial in both human and veterinary medicine [63,64,65]. According to European Network for Optimization of Veterinary Antimicrobial Treatment (ENOVAT) definitions, perioperative antimicrobial prophylaxis refers to antimicrobial administration from 2 h before the surgical incision until 24 h after wound closure, while postoperative antimicrobials are defined as those initiated beyond that 24 h window. In our study, all dogs received perioperative prophylaxis (cefazolin at induction and, when required, intraoperatively), and 94% also received postoperative antibiotics. Despite this extensive use, the observed SSI rate was comparable to that reported in cohorts not receiving antibiotics [11]. Several studies have reported limited or no benefit from perioperative [12,17,64,66] or postoperative [14,67] antibiotic use in clean procedures. In one retrospective cohort, postoperative antibiotics were even associated with increased complication odds [26]. However, only 6% of procedures in our study were performed without postoperative antibiotics, which limits the ability to assess their specific contribution to SSI risk. Further prospective studies with balanced exposure and standardized surveillance protocols are needed to clarify the role of extended antimicrobial use in clean soft tissue surgeries such as mastectomy.

The primary limitation of this study lies in its retrospective design, which inherently depends on the completeness and accuracy of clinical records. Although the standardized application of CDC criteria for diagnosing and classifying SSI strengthens the methodological rigor compared to previous reports, the use of passive rather than active surveillance may have underestimated the true incidence. This potential bias was mitigated through strict case selection and adherence to a standardized, structured follow-up protocol established by the Mammary Oncology Unit of our institution. Nonetheless, prospective studies employing active surveillance strategies are warranted to validate and expand upon these results.

## 5. Conclusions

In summary, the incidence of SSI following canine mastectomy is comparable to that observed in other clean surgical procedures. This study identifies prolonged anaesthetic duration and intraoperative hypothermia as significant, modifiable risk factors associated with increased SSI risk. These findings support the implementation of targeted perioperative strategies, such as minimizing anaesthesia time and maintaining intraoperative normothermia, to reduce postoperative complications. Additionally, neither the surgical extent nor the use of wound soaker catheters influenced SSI risk, reinforcing the safety of incorporating these catheters into multimodal analgesic protocols. These findings reinforce the importance of perioperative care in minimizing postoperative complications in dogs undergoing mastectomy for mammary tumours.

## Figures and Tables

**Table 1:** Perioperative variables of the surgical procedures.

General Variables of the Surgical Procedures		
Variable	N	%
Concurrent disease		
No	351	93.4
Yes	25	6.6
Endocrine disease		
No	371	98.7
Yes	5	1.3
Skin disease		
No	365	97.1
Yes	11	2.4
Leishmaniasis		
No	367	97.3
Yes	9	2.4
Kidney disease		
No	366	97.3
Yes	10	2.7
Anaemia		
No	363	97.8
Yes	8	2.2
Leukopenia		
No	367	98.9
Yes	4	1.1
Leucocytosis		
No	367	98.9
Yes	4	1.1
Thrombocytosis		
No	360	97.0
Yes	11	3.0
Thrombocytopenia		
No	367	98.9
Yes	4	1.1
Blood Proteins		
Normal	351	94.1
Hypoproteinaemia	4	1.1
Hyperproteinaemia	18	4.8
Total	373	100
Hyperglycaemia		
No	364	99.5
Yes	2	0.5
Total	366	100
Hypoglycaemia		
No	366	100
Yes	0	0.0
Total	366	100
Hypoalbuminemia		
No	364	99.5
Yes	2	0.5
Total	366	100
Azotaemia		
No	363	99.2
Yes	3	0.8
Total	366	100
**Surgical procedure variables**		
**Variable**	**N**	**%**
Type of mastectomy		
Nodulectomy	26	7.0
Regional mastectomies	155	41.6
Radical mastectomies	192	51.5
Reproductive status		
Previous sterilization	155	41.6
Concurrent sterilization	218	58.4
Number of mammary tumours		
0	1	0.3
1	112	31.0
2	96	26.6
3	59	16.3
4	35	9.7
5	21	5.8
6	27	7.5
7	5	1.4
8	1	0.3
9	3	0.8
12	1	0.3
Anaesthetic block		
No	255	70.6
Yes	106	29.4
Type of block		
Epidural	48	45.3
Tap-block	58	54.7
Hypotension		
No	247	67.5
Yes	119	32.5
Hypothermia		
No	320	87.4
Yes	46	12.6
**Surgical variables of the surgical procedures**		
**Variable**	**Median (25th–75th Percentile)**	**N**
Maximum size of each nodule		
	1.5 (0.6–3.0)	323
**Variable**	**Mean (SD)**	**N**
Duration of anaesthesia		
	173.5 (54.7)	346
Duration of surgery		
	106 (45.8)	346
Minimum temperature during surgery		
	36.1 (1.0)	323
**Postoperative variables of the surgical procedures**		
**Variable**	**N**	**%**
Postoperative complications		
No	251	69.0
Yes	113	31.0
Seroma		
No	292	88.0
Yes	40	12.0
Dehiscence		
No	331	99.1
Yes	3	0.9
Haematoma		
No	321	86.5
Yes	50	13.5
Necrosis		
No	368	98.9
Yes	4	1.1
Subcutaneous emphysema		
No	370	99.7
Yes	1	0.3
Pain		
No	357	96.0
Yes	15	4.0
Vomit		
No	353	94.9
Yes	19	5.1
Diarrhoea		
No	365	98.4
Yes	6	1.6
Postoperative antibiotic administration		
No	22	6.3
Yes	327	93.7
Clavulanate amoxicillin	210	60.5
Cefazolin	109	31.4
Marbofloxacin	6	1.7
Postoperative anti-inflammatory treatment		
No	12	6.4
Yes	352	93.6
Meloxicam	83	23.6
Carprofen	239	67.9
Robenacoxib	30	8.5
Histological type of tumour		
Benign	192	51.1
Malignant	184	48.9
Grade of malignancy		
I	117	31.9
II	39	10.4
III	25	6.6
**Surgical site infection of the surgical procedures**		
**Variable**	**N**	**%**
Presence of SSI		
No	343	91.2
Yes	33	8.8
Type of SSI		
SSI superficial	31	93.9
SSI deep	2	6.1

**Table 2:** Multivariate logistic regression model of the surgical procedures.

Variables	OR (CI 95%)	*p*
**Median duration of anaesthesia**		
≤173.53	1	
>173.53	2.2 (1–5.2)	**0.041**
**Hypothermia**		
No	1	
Yes	3.1 (1.1–8.3)	**0.026**
Hosmer–Lemeshow test, *p* = 0.888 and area under the ROC curve: 0.657; 95% CI: 0.564–0.750		

## Data Availability

The original contributions presented in this study are included in the article. Further inquiries can be directed to the corresponding authors.

## Abbreviations

The following abbreviations are used in this manuscript:

SSI	Surgical site infection
CDC	Centers for Disease Control
WSCs	Wound soaker catheters
VTH	Veterinary Teaching Hospital
MOU	Mammary Oncology Unit
ASA	American Society of Anesthesiologists
AUC	Area under the curve

## Appendix A

### Appendix A.1. Clinical Management Protocol for Dogs, Mammary Oncology Unit (MOU), Veterinary Teaching Hospital, Complutense University of Madrid

The clinical management of female dogs diagnosed at the Mammary Oncology Unit (MOU) of the Veterinary Teaching Hospital (Complutense University of Madrid) followed a standardized multidisciplinary protocol developed by the MOU. This protocol encompassed all stages of care—from initial evaluation to long-term monitoring—and was applied consistently to ensure high standards of oncological and surgical care.

#### Appendix A.1.1. Preoperative Evaluation

Prior to surgery, all patients underwent a comprehensive anamnesis, including detailed reproductive history (spay status and timing, number of litters, oestrous cycle regularity, and any prior hormonal treatments). Special attention was paid to signs of pseudopregnancy or pseudolactation. A complete physical examination was performed, with systematic palpation of the mammary chains and regional lymph nodes. Each palpable nodule was documented with respect to its anatomical location, three-dimensional size, adherence to adjacent tissues, ulceration, and presence of secretion. Fine-needle aspiration cytology was carried out when lymphadenomegaly was identified.

All patients underwent standard diagnostic imaging, including three-view thoracic radiographs. Abdominal ultrasound was additionally performed in cases with regional lymph node involvement or tumours suspected to be large-volume (T3). A complete blood count, serum biochemistry profile, and preoperative electrocardiogram (ECG) were also obtained as part of the routine preoperative work-up.

#### Appendix A.1.2. Tumour Staging

Tumours were classified using the TNM system adapted for canine mammary neoplasms, incorporating tumour size (T), regional lymph node involvement (N), and presence or absence of distant metastasis (M). The size categories were adjusted according to body weight. In dogs under 10 kg, T1 corresponded to tumours < 2 cm, T2 to 2–3 cm, and T3 to >3 cm. In dogs over 10 kg, T1 included tumours < 3 cm, T2 those measuring 3–5 cm, and T3 those > 5 cm. Lymph node status was categorized as N0 (no metastasis) or N1 (confirmed metastasis), and distant metastasis as M0 (absent) or M1 (present).

Based on these variables, tumours were assigned to five clinical stages: Stage I (T1 N0 M0), Stage II (T2 N0 M0), Stage III (T3 N0 M0), Stage IV (any T, N1, M0), and Stage V (any T, any N, M1).

#### Appendix A.1.3. Surgical Planning and Procedures

Surgical planning was determined by tumour staging and clinical presentation. Nodulectomy was reserved for small, well-defined tumours (<5 mm) located peripherally within the mammary gland in selected Stage I cases. Simple mastectomy was generally indicated for isolated Stage I tumours where surgical margins could be confidently achieved. Regional mastectomy was the most commonly employed technique, used in Stage II cases as well as in selected Stage I tumours requiring wider resection. Radical mastectomy was reserved for multifocal tumours, large or invasive T3 masses, and advanced clinical stages (III and IV). In cases of non-resectable or metastatic disease (Stage V), palliative surgery was considered.

When multiple mammary glands were affected or bilateral disease was present, surgical procedures were staged. Each side was operated separately with a minimum interval of one to two months to allow for appropriate tissue healing and patient recovery. All surgeries were performed or directly supervised by board-certified or nationally accredited veterinary surgeons. In this cohort, all procedures beyond simple mastectomy were classified as major soft tissue surgeries.

#### Appendix A.1.4. Immediate Postoperative Care and Monitoring

Following surgery, all dogs remained hospitalized for a minimum of 24 h to allow for clinical monitoring and immediate postoperative care. During this period, pain control, wound integrity, and systemic recovery were closely assessed. After discharge, patients were re-evaluated on two scheduled occasions: between postoperative days 3 and 5, and again between days 12 and 15. These assessments included inspection of the surgical site, evaluation of pain and general condition, and review of any complications. Skin sutures or staples were typically removed during the second visit, between days 12 and 21, depending on the rate of healing. Additional rechecks were scheduled on a case-by-case basis in response to postoperative complications or delayed wound healing.

#### Appendix A.1.5. Histopathology

All excised mammary tumours and associated lymph nodes were submitted for histopathological examination. Diagnoses were established by board-certified veterinary pathologists, who also provided tumour classification and histological grading when applicable. These findings informed the postoperative staging and follow-up plan and were integrated into the patient’s medical record.

#### Appendix A.1.6. Long-Term Follow-Up

In dogs diagnosed with at least one malignant tumour, a structured long-term follow-up protocol was implemented. Follow-up continued for up to two years after surgery, with evaluations scheduled every three months. Each visit included a complete clinical examination, with particular attention to the surgical site, regional lymph nodes, and thorough palpation of the remaining mammary glands. Thoracic radiographs were performed every three months in all dogs in which a malignant neoplasm had been identified; abdominal ultrasound was performed routinely in dogs with high-grade malignant tumours or dogs with confirmed lymph node metastases in the histopathology. Blood tests were also carried out when necessary to monitor potential adverse effects in those dogs requiring adjuvant postsurgical treatment, depending on the drugs used. This comprehensive follow-up plan aimed to detect early signs of recurrence and guide additional treatment when needed.

**Table A1:** Frequency of patient breeds.

Breed	N (*n* = 306)	%
Crossbreed	98	32.0
Yorkshire Terrier	37	12.1
German Shepherd	17	5.6
Labrador Retriever	12	4.0
Cocker Spaniel	10	3.3
Maltese	10	3.3
West Highland White Terrier	9	2.9
Poodle	9	2.9
Miniature Schnauzer	9	2.9
Beagle	8	2.6
French Bulldog	8	2.6
Weimaraner	8	2.6
Golden Retriever	6	2.1
Shih-tzu	6	2.1
Boxer	5	1.7
Bichon Frise	4	1.4
Chihuahua	3	0.9
Greyhound	3	0.9
Jack Russel Terrier	3	0.9
Belgium Shepherd	3	0.9
Miniature Pinscher	3	0.9
Dachshund	3	0.9
Siberian Husky	3	0.9
Cairn Terrier	2	0.7
Fox Terrier	2	0.7
Briard	2	0.7
Basque Shepherd	2	0.7
Pekingese	2	0.7
Portuguese Podengo	2	0.7
Ratonero Bodeguero Andaluz	2	0.7
Shar Pei	2	0.7
Brittany Spaniel	2	0.7
Bearded Collie	1	0.3
Bull Terrier	1	0.3
English Bulldog	1	0.3
Pug	1	0.3
Cavalier King Charles Spaniel	1	0.3
Catalan Sheepdog	1	0.3
Irish Terrier	1	0.3
Spanish Mastiff	1	0.3
Pomeranian	1	0.3
Staffordshire Bull Terrier	1	0.3
Whippet	1	0.3

**Table A2:** Univariate logistic regression model of the surgical procedures.

General and Clinical Variables of the Surgical Procedures						
Variables	No Infection		Infection		OR (CI 95%)	*p*
	*n*	%	*n*	%		
Weight (kg)						
≤10 kg	178	93.7	12	6.3	1	
>10 kg	165	88.7	21	11.3	1.8 (0.9–3.9)	0.092
Body Score Condition (1/9)						
<5 out of 9	188	90.0	21	10.0	1	
≥5 out of 9	106	91.4	10	8.6	0.8 (0.3–1.8)	0.675
Age (years)						
≤10 years	170	90.9	17	9.1	1	
>10 years	173	91.5	16	8.5	0.9 (0.4–1.8)	0.830
Concurrent disease						
No	321	91.5	30	8.5	1	
Yes	22	88.0	3	12	1 (0.9–1.2)	0.391
Endocrine disease						
No	338	91.1	33	8.9	1	
Yes	5	100.0	0	0.0	0 (0–0)	0.999
Skin disease						
No	333	91.2	32	0.1	1	
Yes	10	90.0	1	0.1	1 (0.1–8.3)	0.970
Leishmaniasis						
No	336	91.6	31	0.1	1	
Yes	7	77.8	2	0.2	3 (0.6–12.5)	0.170
ASA status						
ASA 2	326	91.1	4	8.9	1	
ASA 3	17	91.2	29	8.8	0.9 (0.6–2.7)	0.977
**Surgical variables of the surgical procedures**						
**Variables**	**No Infection**		**Infection**		**OR (CI 95%)**	***p***
	***n***	**%**	***n***	**%**		
Experience of the surgeons						
Senior surgeons	41	91.1	4	8.9	1	
Surgeons-in-training	302	91.2	29	8.8	0.9 (0.6–2.7)	0.977
Type of mastectomy						
Regional mastectomies	140	90.3	15	9.7	1	
Radical mastectomies	175	91.1	17	8.9	1.1 (0.6–2.0)	0.700
Reproductive status						
Previously sterilized	140	90.3	15	9.7	1	
Sterilization performed concurrently	200	91.7	18	8.3	0.8 (0.4–1.7)	0.634
Number of mammary tumours						
≤2	189	90.4	20	9.6	1	
>2	140	92.1	12	7.9	0.810 (0.3–3.4)	0.581
Maximum size of each nodule						
≤1.5	159	92.4	13	7.6	1	
1.5	163	90.1	18	9.9	1.3 (0.6–2.8)	0.430
Duration of anaesthesia						
≤174	161	94.2	10	5.8	1	
>174	152	86.9	23	13.1	2.4 (1.1–5.2)	0.024
Duration of surgery						
≤106	156	91.2	15	8.8	1	
>106	157	89.7	18	10.3	1.1 (0.5–2.4)	0.632
Adhesion to deep plane						
No	304	91	30	9.0	1	
Yes	35	92.1	3	7.9	0.8 (0.2–2.9)	0.823
Ulcer						
No	330	91.2	32	8.8	1	
Yes	9	90.0	1	10.0	1.1 (0.1–9.3)	0.120
Hypotension						
No	226	91.5	21	8.5	1	
Yes	108	90.8	11	9.2	1 (0.5–2.3)	0.814
Hypothermia						
No	103	95.4	5	4.6	1	
Yes	189	87.5	27	12.5	2.9 (1.1–7.8)	0.032
Analgesic diffusion catheters						
No	153	92.2	13	7.8	1	
Yes	190	90.5	20	9.5	0.5 (0.5–2.5)	1.239
**Postoperative variables of the surgical procedures**						
**Variables**	**No Infection**		**Infection**		**OR (CI 95%)**	***p***
	***n***	**%**	***n***	**%**		
Analgesic diffusion catheters						
No	153	92.2	13	7.8	1	
Yes	190	90.5	20	9.5	0.5 (0.5–2.5)	1.239
Postoperative antibiotics						
No	23	88.5	3	11.5	1	
Yes	296	90.8	30	9.2	0.7 (0.2–2.7)	0.695
Malignant tumour						
Benign	176	91.7	16	8.3	1	
Malignant	167	91.2	17	8.8	0.8 (0.2–3.1)	0.654
Histopathological malignancy grade						
I	109	93.3	8	6.7	1	
II	33	84.6	6	15.4	2 (0.6–6.3)	0.199
III	22	88.0	3	12.0	1.6 (0.4–6.6)	0.710

## References

[1] Egenvall A., Bonnett B.N., Ohagen P., Olson P., Hedhammar A., von Euler H. (2005). Incidence of and Survival after Mammary Tumors in a Population of over 80,000 Insured Female Dogs in Sweden from 1995 to 2002. Prev. Vet. Med..

[2] Merlo D.F., Rossi L., Pellegrino C., Ceppi M., Cardellino U., Capurro C., Ratto A., Sambucco P.L., Sestito V., Tanara G. (2008). Cancer Incidence in Pet Dogs: Findings of the Animal Tumor Registry of Genoa, Italy. J. Vet. Intern. Med..

[3] Moe L. (2001). Population-Based Incidence of Mammary Tumours in Some Dog Breeds. J. Reprod. Fertil. Suppl..

[4] Salas Y., Márquez A., Diaz D., Romero L. (2015). Epidemiological Study of Mammary Tumors in Female Dogs Diagnosed during the Period 2002–2012: A Growing Animal Health Problem. PLoS ONE.

[5] Vascellari M., Capello K., Carminato A., Zanardello C., Baioni E., Mutinelli F. (2016). Incidence of Mammary Tumors in the Canine Population Living in the Veneto Region (Northeastern Italy): Risk Factors and Similarities to Human Breast Cancer. Prev. Vet. Med..

[6] Sorenmo K., Worley D., Zappulli V. (2019). Tumors of the Mammary Gland.

[7] Sorenmo K. (2003). Canine Mammary Gland Tumors. Vet. Clin. N. Am. Small Anim. Pract..

[8] Sorenmo K.U., Kristiansen V.M., Cofone M.A., Shofer F.S., Breen A.-M., Langeland M., Mongil C.M., Grondahl A.M., Teige J., Goldschmidt M.H. (2009). Canine Mammary Gland Tumours; a Histological Continuum from Benign to Malignant; Clinical and Histopathological Evidence. Vet. Comp. Oncol..

[9] Zappulli V., Kiupel M., Peña L., Rasotto R., Goldschmidt M.H., Gama A., Scruggs J.L., Kiupel M. (2018). Surgical Pathology of Tumors of Domestic Animals Volume 2, Mammary Tumors.

[10] Novosad C.A. (2003). Principles of Treatment for Mammary Gland Tumors. Clin. Tech. Small Anim. Pract..

[11] Spare P., Ljungvall I., Ljungvall K., Bergström A. (2021). Evaluation of Post-Operative Complications after Mastectomy Performed without Perioperative Antimicrobial Prophylaxis in Dogs. Acta Vet. Scand..

[12] Beal M.W., Brown D.C., Shofer F.S. (2000). The Effects of Perioperative Hypothermia and the Duration of Anesthesia on Postoperative Wound Infection Rate in Clean Wounds: A Retrospective Study. Vet. Surg..

[13] Brown D.C., Conzemius M.G., Shofer F., Swann H. (1997). Epidemiologic Evaluation of Postoperative Wound Infections in Dogs and Cats. Sci. Rep..

[14] Espinel-Rupérez J., Martín-Ríos M.D., Salazar V., Baquero-Artigao M.R., Ortiz-Díez G. (2019). Incidence of Surgical Site Infection in Dogs Undergoing Soft Tissue Surgery: Risk Factors and Economic Impact. Vet. Rec. Open.

[15] Eugster S., Schawalder P., Gaschen F., Boerlin P. (2004). A Prospective Study of Postoperative Surgical Site Infections in Dogs and Cats. Vet. Surg..

[16] Frey T.N., Hoelzler M.G., Scavelli T.D., Fulcher R.P., Bastian R.P. (2010). Risk Factors for Surgical Site Infection-Inflammation in Dogs Undergoing Surgery for Rupture of the Cranial Cruciate Ligament: 902 Cases (2005–2006). J. Am. Vet. Med. Assoc..

[17] Nicholson M., Beal M., Shofer F., Brown D.C. (2002). Epidemiologic Evaluation of Postoperative Wound Infection in Clean-Contaminated Wounds: A Retrospective Study of 239 Dogs and Cats. Vet. Surg..

[18] Stetter J., Boge G.S., Grönlund U., Bergström A. (2021). Risk Factors for Surgical Site Infection Associated with Clean Surgical Procedures in Dogs. Res. Vet. Sci..

[19] Turk R., Singh A., Weese J.S. (2014). Prospective Surgical Site Infection Surveillance in Dogs: Prospective Surgical Site Infection Surveillance. Vet. Surg..

[20] Vasseur P.B., Levy J., Dowd E., Eliot J. (1988). Surgical Wound Infection Rates in Dogs and Cats Data from a Teaching Hospital. Vet. Surg..

[21] Burgess B.A. (2019). Prevention and Surveillance of Surgical Infections: A Review. Vet. Surg..

[22] Garcia Stickney D.N., Thieman Mankin K.M. (2018). The Impact of Postdischarge Surveillance on Surgical Site Infection Diagnosis. Vet. Surg..

[23] Dacanay S.J., Barber R.M., Diehl K.A., Myrna K.E. (2022). Incidence and Risk Factors for Surgical Site Infection Following Enucleation in Dogs. Front. Vet. Sci..

[24] Husi B.A., Arnaldi L., Roitner M., Nolff M.C. (2023). Retrospective Evaluation of Surgical Site Infection after Open Splenectomies with and without Perioperative Prophylactic Antibiotic Coverage. Tierarztl. Prax. Ausg. K. Kleintiere Heimtiere.

[25] Rigby B.E., Malott K., Hetzel S.J., Soukup J.W. (2021). Incidence and Risk Factors for Surgical Site Infections Following Oromaxillofacial Oncologic Surgery in Dogs. Front. Vet. Sci..

[26] Evans B.J., Holt D.E., Stefanovski D., Sorenmo K.U. (2021). Factors Influencing Complications Following Mastectomy Procedures in Dogs with Mammary Gland Tumors: 140 Cases (2009–2015). J. Am. Vet. Med Assoc..

[27] Horta R.S., Figueiredo M.S., Lavalle G.E., Costa M.P., Cunha R.M.C., Araújo R.B. (2015). Surgical Stress and Postoperative Complications Related to Regional and Radical Mastectomy in Dogs. Acta Vet. Scand..

[28] Abelson A.L., McCobb E.C., Shaw S., Armitage-Chan E., Wetmore L.A., Karas A.Z., Blaze C. (2009). Use of Wound Soaker Catheters for the Administration of Local Anesthetic for Post-Operative Analgesia: 56 Cases. Vet. Anaesth. Analg..

[29] Hardie E.M., Lascelles B.D.X., Meuten T., Davidson G.S., Papich M.G., Hansen B.D. (2011). Evaluation of Intermittent Infusion of Bupivacaine into Surgical Wounds of Dogs Postoperatively. Vet. J..

[30] Radlinsky M.G., Mason D.E., Roush J.K., Pineda R. (2005). Use of a Continuous, Local Infusion of Bupivacaine for Postoperative Analgesia in Dogs Undergoing Total Ear Canal Ablation. J. Am. Vet. Med. Assoc..

[31] Raske M., McClaran J.K., Mariano A. (2015). Short-Term Wound Complications and Predictive Variables for Complication after Limb Amputation in Dogs and Cats. J. Small Anim. Pract..

[32] Wolfe T.M., Bateman S.W., Cole L.K., Smeak D.D. (2006). Evaluation of a Local Anesthetic Delivery System for the Postoperative Analgesic Management of Canine Total Ear Canal Ablation—A Randomized, Controlled, Double-Blinded Study. Vet. Anaesth. Analg..

[33] Correa Valencia N.M., Moreno Velásquez D., Vergara Saldarriaga L.A., Uribe Rendón A. (2019). Difusión analgésica de lidocaína administrada a través de un catéter perilesional en hembras caninas sometidas a mastectomía: Un reporte de caso. CES Med. Vet. Zootec..

[34] Suárez-Redondo M., Fuertes-Recuero M., Guzmán-Soltero A., Aguado D., Del Carmen Martín-Espada M., Espinel-Rupérez J., Ortiz-Diez G. (2024). Description of Postoperative Complications and Bacterial Contamination of Wound Soaker Catheters Used to Administer Postoperative Local Analgesia after Mastectomy in 11 Dogs: Case Series. Vet. Res. Commun..

[35] Pinello K., Pires I., Castro A.F., Carvalho P.T., Santos A., de Matos A., Queiroga F., Niza-Ribeiro J. (2022). Vet-OncoNet: Developing a Network of Veterinary Oncology and Reporting a Pioneering Portuguese Experience. Vet. Sci..

[36] Rodríguez J., Santana Á., Herráez P., Killick D.R., de Los Monteros A.E. (2022). Epidemiology of Canine Mammary Tumours on the Canary Archipelago in Spain. BMC Vet. Res..

[37] Vazquez E., Lipovka Y., Cervantes-Arias A., Garibay-Escobar A., Haby M.M., Queiroga F.L., Velazquez C. (2023). Canine Mammary Cancer: State of the Art and Future Perspectives. Animals.

[38] Arenas C., Peña L., Granados-Soler J.L., Pérez-Alenza M.D. (2016). Adjuvant Therapy for Highly Malignant Canine Mammary Tumours: Cox-2 Inhibitor versus Chemotherapy: A Case-Control Prospective Study. Vet. Rec..

[39] Goldschmidt M., Peña L., Rasotto R., Zappulli V. (2011). Classification and Grading of Canine Mammary Tumors. Vet. Pathol..

[40] Peña L., De Andrés P.J., Clemente M., Cuesta P., Pérez-Alenza M.D. (2013). Prognostic Value of Histological Grading in Noninflammatory Canine Mammary Carcinomas in a Prospective Study with Two-Year Follow-up: Relationship with Clinical and Histological Characteristics. Vet. Pathol..

[41] Rutteman G.R., Withrow S.J., Macewen E.G., Withrow S.J., Macewen E.G. (2001). Tumors of the Mammary Gland. Small Animal Clinical Oncology.

[42] Belo L., Serrano I., Cunha E., Carneiro C., Tavares L., Miguel Carreira L., Oliveira M. (2018). Skin Asepsis Protocols as a Preventive Measure of Surgical Site Infections in Dogs: Chlorhexidine-Alcohol versus Povidone-Iodine. BMC Vet. Res..

[43] Brodeur A., Wright A., Cortes Y. (2017). Hypothermia and Targeted Temperature Management in Cats and Dogs. J. Vet. Emerg. Crit. Care.

[44] Reid J., Nolan A., Hughes J., Lascelles D., Pawson P., Scott E. (2007). Development of the Short-Form Glasgow Composite Measure Pain Scale (CMPS-SF) and Derivation of an Analgesic Intervention Score. Anim. Welf..

[45] Berríos-Torres S.I., Umscheid C.A., Bratzler D.W., Leas B., Stone E.C., Kelz R.R., Reinke C.E., Morgan S., Solomkin J.S., Mazuski J.E. (2017). Centers for Disease Control and Prevention Guideline for the Prevention of Surgical Site Infection, 2017. JAMA Surg..

[46] Horan T.C., Gaynes R.P., Martone W.J., Jarvis W.R., Emori T.G. (1992). CDC Definitions of Nosocomial Surgical Site Infections, 1992: A Modification of CDC Definitions of Surgical Wound Infections. Am. J. Infect. Control.

[47] Kurz A., Sessler D.I., Lenhardt R. (1996). Perioperative Normothermia to Reduce the Incidence of Surgical-Wound Infection and Shorten Hospitalization. Study of Wound Infection and Temperature Group. N. Engl. J. Med..

[48] Cheadle W.G. (2006). Risk Factors for Surgical Site Infection. Surg. Infect..

[49] Garceau C., Cosgrove M.S., Gonzalez K. (2023). Inadvertent Perioperative Hypothermia. AANA J..

[50] Simegn G.D., Bayable S.D., Fetene M.B. (2021). Prevention and Management of Perioperative Hypothermia in Adult Elective Surgical Patients: A Systematic Review. Ann. Med. Surg..

[51] Thieman Mankin K.M., Cohen N.D. (2020). Randomized, Controlled Clinical Trial to Assess the Effect of Antimicrobial-Impregnated Suture on the Incidence of Surgical Site Infections in Dogs and Cats. J. Am. Vet. Med. Assoc..

[52] Cruse P.J., Foord R. (1980). The Epidemiology of Wound Infection. A 10-Year Prospective Study of 62,939 Wounds. Surg. Clin. N. Am..

[53] Salo M. (1992). Effects of Anaesthesia and Surgery on the Immune Response. Acta Anaesthesiol. Scand..

[54] Saxena S., Krombach J.W., Nahrwold D.A., Pirracchio R. (2020). Anaesthesia-Specific Checklists: A Systematic Review of Impact. Anaesth. Crit. Care Pain Med..

[55] Razavi B.M., Fazly Bazzaz B.S. (2019). A Review and New Insights to Antimicrobial Action of Local Anesthetics. Eur. J. Clin. Microbiol. Infect. Dis..

[56] Bristow P.C., Halfacree Z.J., Baines S.J. (2015). A Retrospective Study of the Use of Active Suction Wound Drains in Dogs and Cats. J. Small Anim. Pract..

[57] Charlesworth T., Sampaio E. (2023). Effect of Hospitalisation on the Rate of Surgical Site Infection in Dogs with Penrose Drains. J. Small Anim. Pract..

[58] Lu H.Y., Wright T.F. (2023). Evaluation of Complications and Long-Term Outcomes Associated with 101 Dogs and Cats Discharged with and without Subcutaneous Active Closed-Suction Drains (2014–2022). J. Am. Vet. Med. Assoc..

[59] Chilson T.R., Chan F.D., Lonser R.R., Wu T.M., Aitken D.R. (1992). Seroma Prevention after Modified Radical Mastectomy. Am. Surg..

[60] Pavletic M. (2018). Tension-Relieving Techniques. Atlas of Small Animal Wound Management and Reconstructive Surgery.

[61] Stokes R., Wustefeld-Janssens B.G., Hinson W., Wiener D.J., Hollenbeck D., Bertran J., Mickelson M., Chen C.L., Selmic L., Aly A. (2023). Surgical and Oncologic Outcomes in Dogs with Malignant Peripheral Nerve Sheath Tumours Arising from the Brachial or Lumbosacral Plexus. Vet. Comp. Oncol..

[62] Franca A., Stamenova P., Thompson J.L. (2024). Histopathological Diagnosis and Surgical Complications Following Bilateral Anal Sacculectomy for the Treatment of Unilateral Canine Apocrine Gland Anal Sac Adenocarcinoma: 35 Cases (2019–2023). J. Small Anim. Pract..

[63] Mangram A.J., Horan T.C., Pearson M.L., Silver L.C., Jarvis W.R. (1999). Guideline for Prevention of Surgical Site Infection. Infect. Control. Hosp. Epidemiol..

[64] Sørensen T.M., Scahill K., Ruperez J.E., Olejnik M., Swinbourne F., Verwilghen D.R., Nolff M.C., Baines S., Marques C., Vilen A. (2024). Antimicrobial Prophylaxis in Companion Animal Surgery: A Scoping Review for European Network for Optimization of Antimicrobial Therapy (ENOVAT) Guidelines. Vet. J..

[65] Weese J.S. (2006). Investigation of Antimicrobial Use and the Impact of Antimicrobial Use Guidelines in a Small Animal Veterinary Teaching Hospital: 1995–2004. J. Am. Vet. Med. Assoc..

[66] Budsberg S.C., Torres B.T., Sandberg G.S. (2021). Efficacy of Postoperative Antibiotic Use after Tibial Plateau Leveling Osteotomy in Dogs: A Systematic Review. Vet. Surg..

[67] Stine S.L., Odum S.M., Mertens W.D. (2018). Protocol Changes to Reduce Implant-Associated Infection Rate after Tibial Plateau Leveling Osteotomy: 703 Dogs, 811 TPLO (2006–2014). Vet. Surg..

